# Maximum mutational robustness in genotype–phenotype maps follows a self-similar blancmange-like curve

**DOI:** 10.1098/rsif.2023.0169

**Published:** 2023-07-26

**Authors:** Vaibhav Mohanty, Sam F. Greenbury, Tasmin Sarkany, Shyam Narayanan, Kamaludin Dingle, Sebastian E. Ahnert, Ard A. Louis

**Affiliations:** ^1^ Rudolf Peierls Centre for Theoretical Physics, University of Oxford, Oxford, UK; ^2^ Program in Health Sciences and Technology, Massachusetts Institute of Technology, Cambridge, MA, USA; ^3^ MD-PhD Program, Harvard Medical School, Boston, MA, USA and Massachusetts Institute of Technology, Cambridge, MA, USA; ^4^ Theory of Condensed Matter Group, Cavendish Laboratory, University of Cambridge, Cambridge, UK; ^5^ Wellcome-MRC Cambridge Stem Cell Institute, University of Cambridge, Cambridge, UK; ^6^ Department of Chemical Engineering and Biotechnology, Cavendish Laboratory, University of Cambridge, Cambridge, UK; ^7^ The Alan Turing Institute, British Library, London, UK; ^8^ Department of Electrical Engineering and Computer Science, Massachusetts Institute of Technology, Cambridge, MA, USA; ^9^ Department of Mathematics and Natural Sciences, Centre for Applied Mathematics and Bioinformatics (CAMB), Gulf University of Science and Technology, Kuwait; ^10^ Department of Computing and Mathematical Sciences, California Institute of Technology, Pasadena, CA, USA

**Keywords:** genotype–phenotype maps, mutational robustness, phenotype coarse-graining, blancmange curve, Trollope–Delange, Takagi

## Abstract

Phenotype robustness, defined as the average mutational robustness of all the genotypes that map to a given phenotype, plays a key role in facilitating neutral exploration of novel phenotypic variation by an evolving population. By applying results from coding theory, we prove that the maximum phenotype robustness occurs when genotypes are organized as bricklayer’s graphs, so-called because they resemble the way in which a bricklayer would fill in a Hamming graph. The value of the maximal robustness is given by a fractal continuous everywhere but differentiable nowhere sums-of-digits function from number theory. Interestingly, genotype–phenotype maps for RNA secondary structure and the hydrophobic-polar (HP) model for protein folding can exhibit phenotype robustness that exactly attains this upper bound. By exploiting properties of the sums-of-digits function, we prove a lower bound on the deviation of the maximum robustness of phenotypes with multiple neutral components from the bricklayer’s graph bound, and show that RNA secondary structure phenotypes obey this bound. Finally, we show how robustness changes when phenotypes are coarse-grained and derive a formula and associated bounds for the transition probabilities between such phenotypes.

## 1. Introduction

A single *genotype* is a collection of biological information. It can be encoded by a sequence of DNA or RNA or in a more coarse-grained way, for example, in the weights of a gene regulatory network. A genotype is mapped to a *phenotype*, which is a biologically observed output, trait or behaviour, via a genotype–phenotype (GP) map [1–3]. Examples include four letter RNA sequences and 20 letter protein sequences that can be mapped to their physical folded states, and gene-regulatory networks, which can, for example, be described by Boolean networks [4] where a set of weights represent the gene interaction strengths.

Because many mutations are effectively neutral there will typically be many more genotypes than phenotypes; for instance, in the RNA secondary structure GP map, for nucleotide sequences of length *L*, there are approximately 1.8^*L*^ phenotypes while there are exactly 4^*L*^ genotypes [5]. The set of all genotypes that map to a given phenotype is called a neutral set, or sometimes also a neutral network. It plays an important role in shaping the way that novel variation arises in evolutionary dynamics. Properties of neutral sets have been extensively studied [1–4,6–26]. A number of key features of neutral sets are shared across GP maps [2,3,19]. For example, the size of the neutral sets for different phenotypes typically vary over many orders of magnitude, with a small fraction of the phenotypes taking up the majority of genotypes. Such phenotype bias can strongly affect evolutionary outcomes [15,18,26–32].

Another important shared trait is that neutral sets are typically highly connected by point mutations due to a high average mutational robustness, meaning that they are likely to be fully connected, or percolate. This property hugely enhances the probability that a neutral set can be traversed by single mutational steps, allowing a much larger set of alternative phenotypes to be accessible than one could reach from a single genotype. In this way, enhanced robustness can lead to enhanced evolvability, which is the ability to discover new phenotypes [10,33]. In some cases, the neutral set is split into smaller component networks which are disconnected, for example due to biophysical constraints [8,11,34]. Then it is not the full neutral set, but rather each neutral component that percolates and is easily traversed via point mutations.

The property that we will focus on in this paper is the mutational robustness *ρ*_*p*_ of a phenotype *p*, defined as the average probability that a single character mutation of a genotype mapping to phenotype *p* does *not* change the phenotype *p*. Typically larger neutral sets have higher robustness. For the (3-non-crossing [35]) RNA sequence-to-secondary structure GP map, it has been shown that the distribution of robustness found upon random sampling of sequences accurately predicts the distribution of robustnesses for functional or non-coding RNAs found in nature [36], although for very short strands, naturally occurring RNA are marginally more robust [26]. In other words, for this system, the structure of GP map appears to largely determine the mutational robustness found in nature. Thus studying these more abstract mathematical features of the GP map may directly lead to predictions about naturally occurring phenotypes.

To study *ρ*_*p*_ in a mathematically convenient way, we will use the language of graphs. The entire set of *k*^ℓ^ possible sequences in a GP map with input sequences of length ℓ drawn from an alphabet of *k* characters is representable as a generalization of a hypercube graph called a *Hamming graph* *H*_ℓ,*k*_. Each sequence maps onto a vertex, and two vertices are connected by an edge in *H*_ℓ,*k*_ only if the corresponding sequences differ by a single character. The neutral set of all genotypes mapping to phenotype *p* define a vertex set *V*(*G*_*p*_) on a vertex-induced subgraph (or neutral set) *G*_*p*_ in *H*_ℓ,*k*_. In other words, each vertex represents one of the genotypes in the neutral set. Similarly, the edge set *E*(*G*_*p*_) is defined as the set of all edges between vertices in *G*_*p*_, and represents genotypes that are connected by neutral mutations. The robustness can now be defined as the average degree of *G*_*p*_ divided by ℓ(*k* − 1) such that it is normalized to 0 ≤ *ρ*_*p*_ ≤ 1. Using the relationship between the average degree and the edge-to-vertex ratio, we have a graph-theoretic definition of robustness *ρ*(*G*_*p*_) of the neutral set *G*_*p*_

1.1 $$\rho (G_{p})=\frac{2|E(G_{p})|}{\ell (k-1)|V(G_{p})|}.\;$$

In other words, this definition of the robustness scales with the average number of edges per vertex, normalized so that it is equal to the fraction of possible mutations in the neutral set that are neutral. Relatively high phenotype robustness is important because high robustness has been shown to facilitate navigability of fitness landscapes [37]. To a good approximation, if the frequency of a phenotype *p* is above the threshold *f*_*p*_ > 1/[ℓ(*k* − 1)], then the graph *G*_*p*_, or equivalently, the neutral set or neutral component, should percolate [19], resulting in very high robustness. Even under the assumption of random GP pairings, above the percolation threshold, the number of neutral components within a neutral set is expected to drop precipitously, and the size of the largest neutral component is expected to sharply increase [19], both of which suggest a highly connected neutral component and thereby high robustness.

A null expectation of the robustness predicts that the probability of a mutation being neutral should scale as

1.2 $$\rho_{p}\approx f_{p}\;\text{(random null model)},$$

where the frequency *f*_*p*_ ≡|*V*(*G*_*p*_)|/*k*^ℓ^ is equal to the probability of obtaining *p* when choosing a genotype at random. This scaling holds when genotypes are completely uncorrelated as they would be in a random null model [19] where genotypes are randomly attributed to phenotypes under the constraint that the neutral set sizes are kept fixed. For the typically strong phenotype bias observed in GP maps, this scaling implies that many phenotypes will have a robustness that is too small to allow neutral set graphs to percolate.

However, empirical studies of robustness have consistently shown a log-linear relationship with the frequency of a phenotype

1.3 $$\rho_{p}\approx 1+\frac{\log_{k}f_{p}}{\ell }\gg f_{p}\;\text{(empirical)},$$

for a wide range of GP maps in biology, as well as for similar input–output maps in computer science and physics [2,3,16–25]. The empirically measured robustness is orders of magnitude higher than what is predicted by the random null model. There must therefore be strong correlations between the genotypes mapping to a particular phenotype [19]. One important biological consequence of this empirically measured higher robustness is that, typically, a large fraction of all neutral set graphs in a GP map should percolate, leading to enhanced evolvability.

These empirical results also raise an interesting question that will be the main focus of this paper, namely *What is the upper bound on robustness, and how close are physical GP maps to this bound?* Here, we prove, by applying concepts from coding theory harking back to the 1960s [38,39], that a particular type of graph called *bricklayer’s graphs* [40] are maximally robust.

We then derive explicit expressions for the maximum robustness of bricklayer’s graphs, which by extension gives a maximum on the possible robustness of neutral sets. These expressions are closely related to the well-known sums-of-digits function from number theory. The dominant term scales as 1 + log_*k*_ *f*_*p*_/ℓ, which resembles the empirical scaling of equation (1.3). Next, we numerically confirm that many individual neutral components for the RNA sequence-to-secondary structure GP maps and the hydrophobic-polar (HP) protein folding GP map introduced by Dill [41] can attain the bricklayer’s graph bound exactly.

We prove that if a full neutral set is made up of several neutral components, then the maximal attainable robustness will be below the maximum given by a bricklayer’s graph. By deriving a new property of the sums-of-digits function, we are able to calculate a lower bound on the robustness of a neutral set made up of multiple connected neutral components. We show numerically that the RNA GP map obeys this lower bound.

Lastly, we consider the coarse-graining of phenotypes. We show how robustness and transition probabilities change when multiple phenotypes are combined into compound phenotypes, and use our equations to explain some results about the robustness of these compound phenotypes.

## 2. Bricklayer’s graphs have maximum robustness

The term ‘bricklayer’s graph’ was coined by Reeves *et al.* [40] in an interesting paper where they studied the principal eigenvalue of the subgraph, which is a different measure of phenotype robustness because it also takes into account population structure at steady state [42]. The name arises because they are constructed by repeatedly adding an adjacent vertex in the Hamming graph in a manner resembling the process of laying bricks. While these particular graphs have a much older origin in coding theory, we will use this more recent nomenclature throughout this work. Bricklayer’s graphs are formally defined as follows:

**Definition 2.1.**

Consider a Hamming graph *H*_ℓ,*k*_ in which all *k*^ℓ^ vertices are labelled with integers 0 to *k*^ℓ^ − 1 according to each integer’s base-*k* representation, and two vertices are connected by an edge if the base-*k* representations differ by exactly one character. A **bricklayer’s graph** *G*_*n*,*k*_ is an induced subgraph of a Hamming graph *H*_ℓ,*k*_ containing the first |*V*(*G*_*n*,*k*_)| = *n* vertices, labelled from {0, 1, …, *n* − 1}.

From our definition of the robustness (1.1), it follows that maximizing robustness for a given number of vertices *V*(*G*) (the size of graph *G* that represents the neutral set) is equivalent to maximizing the number of edges of the equivalent graph. Finding the subgraph *G* with a fixed number of vertices of a Hamming graph *H*_ℓ,*k*_ that maximizes the number of edges (and robustness) is equivalent to minimizing the ‘edge boundary’ of the subgraph, i.e. minimizing the number of edges {*u*, *v*} which connect a subgraph vertex *u* ∈ *V*(*G*) to a vertex outside the subgraph $v\in V(H_{\ell ,k}\setminus G)$. This is known as the ‘edge-isoperimetric problem’ for the Hamming graph. This problem is connected to coding theory because, for example, it was effectively proven in [38,39] that if the vertices of a bricklayer’s graph (which they referred to as a vertex numbering which maximizes ‘connectedness’, similar to robustness) represent the set of *k*-ary sequences that map to a particular codeword being transmitted, then this set of sequences minimizes the number of single-site mutations that would cause an incorrect transmission of the codeword, averaged over all the sequences mapping to that codeword. This is akin to maximizing mutational robustness, which measures the average number of point mutations that do *not* change the phenotype, in biological systems. More specifically, Harper [38] showed that bricklayer’s graphs attain the maximum bound for the *k* = 2 case, and Graham [43] and Hart [44] calculated the exact value of the bound for *k* = 2, namely |*E*(*G*)| ≤ *S*_2_(*n*), where $S_{k}(n)=\sum_{i=0}^{n-1}s_{k}(i)$ is formulated in terms of *s*_*k*_(*i*), the sum of all digits in the base-*k* representation of the integer *i*. We will call *S*_*k*_(*n*) the *sums-of-digits function*.

Importantly for this study, Lindsey [39] generalized the work of Harper [38] to prove that bricklayer’s graphs (not necessarily uniquely) attain the maximum bound for all *k* ≥ 2 although he did not calculate the value of the bound. No other graph can have a robustness higher than a bricklayer’s graph, although some graphs may attain the same robustness.

## 3. Robustness of bricklayer’s graphs

### 3.1. Exact robustness/number of edges

To exactly calculate the maximum robustness, we first need to prove a theorem about the maximum number of edges of a bricklayer’s graph.

**Theorem 3.1.**

*A bricklayer’s graph* *G*_*n*,*k*_(*V*, *E*) *with* *n* *vertices has* $\left|E|=S_{k}(n)=\sum_{i=0}^{n-1}s_{k}(i\right)$ *edges, where* *s*_*k*_(*i*) *is the sum of all digits in the base*-*k* *representation of the integer* *i*, *and* *S*_*k*_(*n*) *is the sums-of-digits function*.

**Proof.**

See appendix A. ▪

**Figure 1.**
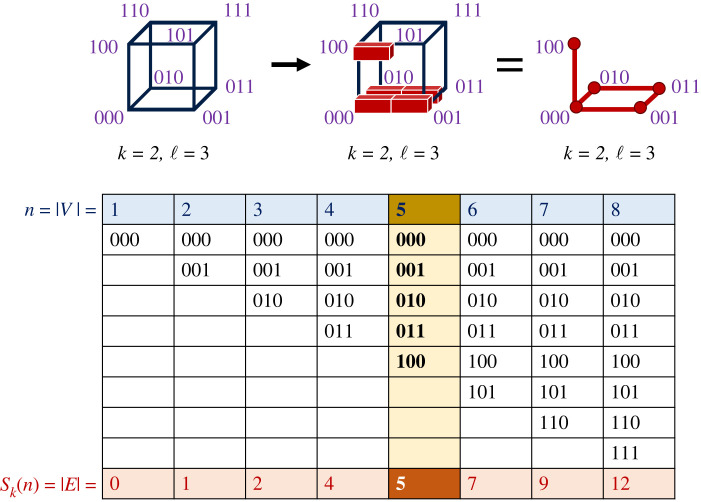
Schematic to illustrate the coding theory/graph theory perspective on the maximum robustness for an induced subgraph of the Hamming graph. (Top) By considering the Hamming graph’s vertices as binary sequences, sequences that differ by exactly one character are connected by an edge. Robustness is proportional to the edge-to-vertex ratio, see equation (1.1). (Top, left) Hamming graph for binary sequences (*k* = 2) of length ℓ = 3, (top, middle) the ‘bricklayer’s graph’ [40] representation of the (top, right) neutral network with maximal robustness for *n* = 5 vertices. (Bottom) Table showing the maximal number of edges for different numbers of vertices, together with an example of a bricklayer graph. In theorem 3.1, |*E*| = *S*_*k*_(*n*) for *k* = 2, making a direct connection between number theory and the maximal number of edges, which is calculated by adding up the digits of the base-*k* representations of the integers 0 to *n* − 1, which are listed in the white rows of the table.

This theorem generalizes the proof by Graham [43] and Hart [44] for all *k* ≥ 2, and improves on the bound given by Squier *et al.* [45]. A graphical example of the theorem and the relationship between the coding/graph theory and number theory perspectives are shown in figure 1.

To calculate the upper bound on robustness, we therefore need to work out the properties of the sums-of-digits function. As far back as 1940, Bush [46] already showed that the asymptotic behaviour (for large *n*) of the sums-of-digits function scaled as *S*_*k*_(*n*) ∼ (*n*/2)(*k* − 1)log_*k*_ *n*. An exact analytical form for *k* = 2 was given by Trollope in 1968 [47] and later generalized by Delange in 1975 for all *k* [48] as

3.1 $$S_{k}(n)=\frac{n}{2}[(k-1)\log_{k}n-g_{k}(k^{\{\log_{k}n\}-1})],$$

where {*x*} denotes the fractional part of *x*, and

3.2 $$g_{k}(x)=(k-1)\log_{k}x+\frac{D_{k}(x)}{x},$$

where *D*_*k*_(*x*) is the *Delange function* (using the modified definition in [49]) given by

3.3 $$\begin{array}{rl} D_{k}(x) & =\sum_{n=0}^{\infty }\frac{D_{k,0}(k^{n}x)}{k^{n}}\\ \mathrm{and}\;D_{k,0}(x) & =\int_{0}^{x}\;dt(2k[t]-2[kt]+k-1), \end{array}\}$$

where [*x*] is the integer part of *x*. For *k* = 2, the Delange function *D*_*k*_(*x*) is the same as the continuous everywhere, differentiable nowhere Takagi function first described in 1903 [50]. The fractal Takagi function is sometimes called the Blancmange function, because it resembles the blancmange dessert. It has many applications, including but not limited to mathematical analysis, probability theory and number theory [51]. The general sums-of-digits function has also interesting connections to many fields, and in particular to number theory. For example, Delange [48] famously showed that the Fourier series coefficients {*c*_*n*_} of *g*_*k*_(*k*^{*x*}−1^) (which is periodic in *x* with a period of one) are defined by

3.4 $$g_{k}(k^{\{x\}-1})=\sum_{n\in Z}c_{n}\;e^{ı2\pi nx}$$

with

3.5 $$c_{n}=ı\frac{k-1}{n\pi }{\left(1+\frac{ı2n\pi }{\log k}\right)}^{-1}\zeta \left(\frac{ı2n\pi }{\log k}\right),$$

which are linked to *ζ*, the Riemann zeta function.

Combining our expression for phenotype robustness (1.1) with theorem 3.1 and equation (3.1) provides an expression for the maximum phenotype robustness for a neutral set of size *n*_*p*_ = |*V*(*G*_*p*_)|

3.6 $$\rho_{p}^{\max}=\frac{2S_{k}(n_{p})}{n_{p}\ell (k-1)}=\frac{\log_{k}n_{p}}{\ell }-\frac{g_{k}(k^{\{\log_{k}n_{p}\}-1})}{\ell (k-1)}.$$

**Figure 2.**
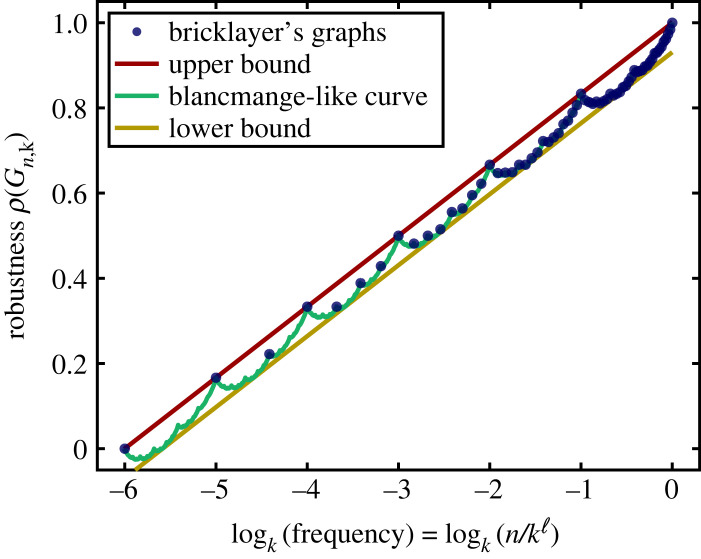
A linear-log plot of the bricklayer’s graphs’ robustness $\rho_{p}^{\max}$ for *n* vertices, given by equation (3.6), versus frequency (number of vertices *n* divided by *k*^ℓ^), where ℓ = 6 and *k* = 2. Each blue dot denotes a possible neutral set size. The green line denotes the continuous everywhere but differentiable nowhere ‘blancmange-like curve’ (here *k* = 2, so one component of this line is exactly equivalent to the Tagaki curve [50]) that is given by the continuous *n*_*p*_ version of equation (3.6), corresponding to $\rho_{\;p}^{\max}$. The upper and lower bounds on $\rho_{\;p}^{\max}$, given by equation (3.8), are also plotted. The upper bound is equivalent to the simple form *ρ*_*p*_ = ℓ^−1^log_*k*_ *n*_*p*_ = 1 + log_*k*_(*f*_*p*_)/ℓ. Plots like this, containing the exact maximum robustness as well as the upper and lower bounds, can be generated with our free, open-source web tool RoBound Calculator [52].

This upper bound is optimal because we can always construct a bricklayer’s graph with *n*_*p*_ vertices. The maximum robustness is plotted in figure 2, and exhibits the expected blancmange-like self-similar form. Since the frequency *f*_*p*_ = *n*_*p*_/*k*^ℓ^, the first term reproduces the empirically observed scaling of equation (1.3), namely *ρ*_*p*_ ≈ ℓ^−1^log _*k*_ *n*_*p*_ = 1 + log_*k*_(*f*_*p*_)/ℓ, which upperbounds the exact robustness in figure 2.

### 3.2. Bounds on neutral set robustness

It would be useful to find simpler expressions to bound the maximum robustness. Indeed, Galkin & Galkina [49] specify the bound

3.7 $$A_{k}\leq \frac{S_{k}(n)}{n}-\frac{k-1}{2}\log_{k}n\leq 0,$$

in terms of a constant *A*_*k*_ that only depends on the alphabet size *k*. This implies that the robustness of a bricklayer’s graph, or equivalently the maximum robustness of a neutral set of size *n* from equation (3.6) is bounded below and above by

3.8 $$\frac{\log_{k}n}{\ell }+\frac{2A_{k}}{(k-1)\ell }\leq \rho_{n}^{\max}\leq \frac{\log_{k}n}{\ell }.$$

In other words, the empirical scaling (1.3) is a strict upper bound to the robustness, and it also provides a lower bound up to terms that scale with O(1/ℓ). We have created a free, open-source web tool called RoBound Calculator using Google Colaboratory which can generate, for specified *k* and ℓ, exact values and continuous interpolations of the bricklayer’s graph robustness $\rho_{n}^{\max}$, as well as the upper and lower bounds from equation (3.8). RoBound Calculator is available at [52], and several example plots from this tool are provided in appendix C.

There is no short formula to calculate *A*_*k*_, but Galkin & Galkina [49] have found an exact, though fairly involved, algorithm to determine *A*_*k*_. For *k* = 2, *A*_2_ = log_4_ 3 − 1 ≈ −0.2075; for *k* = 3, *A*_3_ = log_3_ 2 − 1 ≈ −0.3691 and for *k* = 4, *A*_4_ = (3/4)log_2_ 5 − (9/4) ≈ −0.5086 (biologically relevant, as DNA/RNA have *k* = 4). Additional biologically relevant values of *k* can be calculated using their algorithm. For instance, proteins have *k* = 20 amino acids comprising their primary sequence, and we can use the algorithm to find that $A_{20}=(19\log(84/23)/\log(400))-\frac{355}{46}\approx -3.6097$. Moreover, as *k* → ∞,

3.9 $$A_{k}=-\frac{k}{2}\left[1-\frac{\log\log k}{\log k}+O\left(\frac{1}{\log k}\right)\right].$$

Finally, note that the correction term 2*A*_*k*_/((*k* − 1)ℓ) that appears on the left-hand side of the equation (3.8) is typically quite small compared with the scale of the typical values of *ρ*_*p*_ found for neutral components of these systems. For instance, this correction term has the value −0.0283 for RNA12, and −0.0226 for RNA15. Equation (3.8) is therefore typically a tight bound (see also figure 2). Thus, the maximum robustness can be quite reasonably approximated by simple $\rho_{p}^{\max}\approx \log_{k}(n_{p})/\ell =1+\log_{k}(f_{p})/\ell$ form.

## 4. Neutral components of biological genotype–phenotype maps can attain the bricklayer’s graph bound

We next turn to the question of how close the neutral components of physical GP maps are to the upper bound (3.6). We study the RNA (length 12 and 15) secondary structure models with all four nucleotides (ACUG) as well as the HP protein folding models (length 24, and 5 × 5 lattice). The RNA and HP simulation data were obtained from prior work [19].

A neutral set for a phenotype may not fully percolate, but it can be broken into multiple neutral components of various sizes that do percolate [19] (though some phenotypes may have only a single component). By definition, a neutral component has no single mutation connections to any other neutral component of that same phenotype’s neutral set.

**Figure 3.**
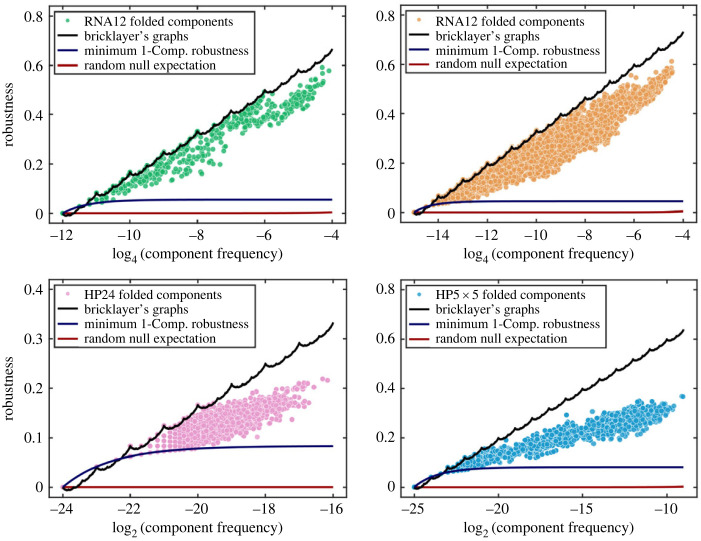
The robustness of every component of every folded phenotype for both the RNA and HP GP maps (of various lengths) is plotted against the frequency (fraction of vertices *f* = |*V*(*G*)|/*k*^ℓ^ in the entire Hamming graph) alongside the continuous interpolation of the maximum robustness curve from equation (3.6)) (black line). The minimum robustness of a neutral component, equation (4.1), is given by the blue line, and the random null expectation, equation (1.2), is plotted as the red line. Note that for these small neutral components, the minimum component robustness exceeds the random null expectation since, by definition, the neutral component consists of vertices which are a connected component. Such a collection of vertices will naturally have more edges than the average collection of vertices randomly selected to form a neutral network in the random null model. The data for these curves can be generated by using the RoBound Calculator [52] tool we have introduced. The natural GP maps all contain neutral components which attain the bricklayer’s graph bound (as well as some very low robustness components that attain the minimum bound). The unfolded (trivial) phenotype is omitted from each of these plots. The minimum robustness appears to be larger than the bricklayer’s graph line for low frequencies; but, this only happens for non-integer values of the number of vertices. Of course, any graph will have an integer number of vertices; in all of those cases, the bricklayer’s graph robustness will be greater than or equal to the minimum robustness.

In figure 3, the robustness values of each neutral component in the RNA12, RNA15, HP24 and HP5 × 5 models are plotted against the logarithm of the number of vertices in that neutral component. For all of the systems studied, many neutral components over several orders of magnitude of phenotype frequencies *f*_*p*_ exactly attain the same robustness as bricklayer’s graphs. Typically, this is more common for smaller than for larger neutral components. In the HP model GP maps, the size of the largest neutral components that still reach the bricklayer’s graph line have fewer vertices than the same for RNA secondary structure GP maps. This is probably due to the architecture of the GP maps themselves; it has been shown recently [53] that neutral components are often modular in that they consist of highly packed clusters of vertices which are then connected to other clusters by a smaller set of linking vertices. We speculate that in the HP model GP map there may be higher modularity, leading to less-than-maximally robust neutral components above a lower threshold. It is also worth mentioning that the RNA and HP systems examined here are fairly small in length because it is computationally quite expensive to exhaustively check the neutral components for much larger sequence lengths.

Figure 3 also depicts the minimum robustness of each fully connected neutral component *G*_*i*_

4.1 $$\rho_{\min}(G_{i})=\frac{2}{\ell (k-1)}\left(1-\frac{1}{\left|V(G_{i})\right|}\right).$$

The above formula follows from the fact that a neutral component, by definition, is connected, and the minimum number of edges in a connected graph with *n* vertices is *n* − 1. This minimum value can be attained by many graphs, including the path graph *P*_*n*_ and star graph *K*_1,*n*_. This is also the robustness that individual phenotypes/outputs have for the one-dimensional Edwards–Anderson spin glass input–output map [25]. Note that the null-model for robustness, *ρ*_*p*_ ≈ *f*_*p*_ includes many disconnected components, and so can be much lower than equation (4.1), which holds for a fully connected component. The RoBound Calculator tool [52] we have introduced also calculates and plots the *ρ*_min_ curve as well.

The strict upper bound (3.6) is close to empirical scaling (1.3) observed for many GP maps, and quite far from the naive scaling *ρ*_*p*_ ≈ *f*_*p*_. Since the actual robustness cannot be higher than the bound, this suggests that, at least on the scale given by *f*_*p*_, physical GP maps exhibit a robustness that is close to the maximum value attainable. This empirical observation raises interesting theoretical questions because the upper bound is not imposed by the specific properties of an individual GP map (biological or not); rather, the robustness is bounded above due to very general mathematical properties of the Hamming graph underlying the GP map.

## 5. Robustness of full neutral sets and the bricklayer’s bound

A bricklayer’s graph is maximally dense in its edge-to-vertex ratio; it must certainly be fully connected. Therefore, if a neutral set is not fully connected, and thus broken down into neutral components, then the full phenotype robustness *ρ*_*p*_ must deviate from this optimum, even if the robustness of each of its components attains the optimal bound.

To calculate a bound on *how much* phenotypes broken down into neutral components deviate from the optimal robustness, we consider a neutral set that has *n* vertices and is split into *m* neutral components. If each neutral component is maximally robust (as many of the RNA/HP neutral components are), then each robustness would be

5.1 $$\rho (G_{n_{i},k})=\frac{2S_{k}(n_{i})}{n_{i}\ell (k-1)},\;1\leq i\leq m,$$

where $n=\sum_{i=1}^{m}n_{i}$, with *n*_*i*_ being the number of vertices in the *i*th neutral component. In other words, the total robustness *ρ*_*p*_ for this specific case of a neutral set made up of bricklayer’s graph components is simply a frequency-weighted sum of the robustnesses of the individual neutral components

5.2 $$\rho_{p}^{m}=(1/n)\sum_{i=1}^{m}n_{i}\rho (G_{n_{i},k})\leq \rho_{p}^{\max},$$

where the last inequality simply follows from the fact that $\rho_{p}^{\max}$ is the strict upper bound on robustness, which can only be obtained when the entire neutral set only has one single component.

To calculate a more accurate bound, we first prove an interesting property of the sums-of-digits function *S*_*k*_(*n*), generalizing the proof by Graham [43], who proved the following for *k* = 2, which we now prove for general *k*

**Theorem 5.1.**

*For* *k* *non-negative integers* {*n*_1_, *n*_2_, …, *n*_*k*_} *obeying* 0 ≤ *n*_1_ ≤ *n*_2_ ≤ · · · ≤ *n*_*k*_, *the following property of the sums-of-digits function holds*:

5.3 $$\sum_{i=1}^{k}S_{k}(n_{i})+\sum_{i=1}^{k-1}(k-i)n_{i}\leq S_{k}\left(\sum_{i=1}^{k}n_{i}\right)$$

**Proof.**

See appendix A. ▪

Theorem 5.1 is not only an interesting property of the sums-of-digits function which may be useful in coding theory, but it also can be used to provide, for the specific case that there are exactly *k* neutral components, a tight bound

5.4 $$\left(\rho (G_{n,k})-\frac{1}{n}\sum_{i=1}^{k}n_{i}\rho (G_{n_{i},k})\right)\geq \frac{2}{n\ell (k-1)}\sum_{i=1}^{k-1}(k-i)n_{i}$$

on the difference between the maximum phenotype robustness for a fully connected neutral set of size *n*, given by equation (6.1) and $\rho_{p}^{k}=(1/n)\sum_{i=1}^{k}n_{i}\rho (G_{n_{i},k})$, the robustness of a neutral set consisting of *k* bricklayer’s graphs as neutral components. A further generalization and discussion of this formula is provided in §6.

The assumption that the neutral components are bricklayer’s graphs is not necessary, however. Weakening this assumption to assume that each neutral component has an arbitrary topology simply weakens the tightness of the bound. For an arbitrary neutral component $A_{n_{i}}$ with *n*_*i*_ vertices, $\rho (A_{n_{i}})\leq \rho (G_{n_{i},k})$, so

5.5 $$\left(\rho (G_{n,k})-\frac{1}{n}\sum_{i=1}^{k}n_{i}\rho (A_{n_{i}})\right)\geq \frac{2}{n\ell (k-1)}\sum_{i=1}^{k-1}(k-i)n_{i}.$$

**Figure 4.**
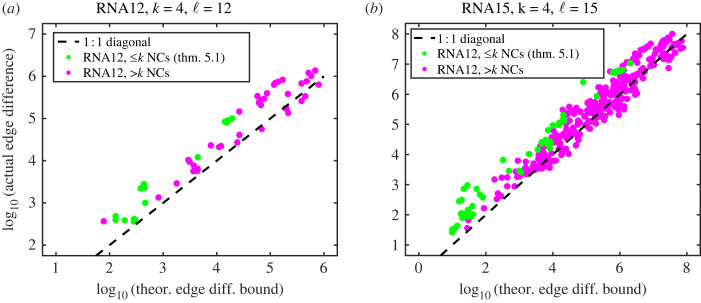
Plot of left-hand side (ordinate) and right-hand side (abscissa) of equation (5.5) for physical phenotype data for RNA12 and RNA15 from our dataset from [19]. Green plot points represent phenotypes with ≤*k* = 4 neutral components; the theoretical bound in equation (5.5) rigorously holds for these phenotypes. Magenta plot points have greater than 4 neutral components; despite the fact that the bound should not rigorously hold for such phenotypes, it still does seem to approximately hold for many phenotypes, or at least many plot points lie close to the dashed line.

It is important to note that this inequality has been proven to hold only when the number of neutral components is less than or equal to *k*. Indeed, it does hold perfectly for the biological RNA neutral component/phenotype robustness data from our dataset from [19]. In figure 4, each plot point represents a phenotype, and the vertical axis coordinate is given by the log of the left-hand side of equation (5.5), which is (the log of) the difference in the optimal number of edges and the actual number of edges for that phenotype. The horizontal axis coordinate is given by the log of the right-hand side of equation (5.5), which is a theoretical bound computed from the frequencies of the neutral components for that phenotype. The figure 4*a* shows actual RNA12 phenotype data, and figure 4*b* RNA 15 phenotype data. Green plot points have *k* = 4 or fewer neutral components; it is for these phenotypes that the theoretical bound rigorously holds. The biological data support the theory if all green plot points are *above or on* the dashed 1 : 1 diagonal line, as this would indicate that the inequality is valid; indeed that is the case.

Most of the phenotypes in the RNA12 and RNA15 secondary structure GP maps do not have ≤*k* = 4 neutral components, however. Also in figure 4, we have plotted, in magenta, the values of the left- and right-hand side of equation (5.5) for phenotypes with >*k* = 4. The theoretical bound seems to hold even for most of the cases outside the range for which it is proven, but it begins to fail for sufficiently large phenotypes.

Here, we discussed the process by which many neutral components are combined into one larger phenotype; these neutral components are not connected to each other (by definition), so the phenotype robustness is simply a frequency-weighted average of the component robustnesses that will be necessarily lower than the maximum possible achievable robustness for a single-component phenotype. With this being said, in practice, an evolving population will typically be confined to a component, as double neutral mutations are typically quite rare. Therefore, even though the robustness of the phenotype is lower, the robustness experienced in shorter time scales by the population may often be closer to the bricklayer’s graph bound.

## 6. Robustness of coarse-grained outputs/phenotypes

In discussing neutral set and neutral component topologies for natural systems, one should keep in mind that the definition of a phenotype can vary depending on which biological property one is interested in. Working out exactly how to map genotypes to phenotypes in biological systems can be difficult due to questions of how a phenotype is defined. For RNA structures, for example, one may be interested in a specific secondary structure, as we are here, or in a more restricted phenotype, such as a certain tertiary structure. Conversely, one may be interested in a broader class of secondary structures or in a different property, such as a certain catalytic function. It is therefore interesting to ask how robustness changes if one zeroes in on different levels of description of a phenotype.

In this section, we will study the robustness of a phenotype and transition probabilities between phenotypes that have been generated from the union of multiple neutral sets. We refer to this process of merging neutral sets of different phenotypes as ‘coarse-graining’ of phenotypes. Tackling this question requires a more generalized approach than was used in the previous section because different phenotypes typically have non-zero transition probabilities between each other, unlike neutral components which have zero transition probabilities (on single mutations).

We illustrate our approach for RNA secondary structure using a coarse-graining method from Giegerich *et al.* [54], who defined a set of new ‘abstract shape’ RNA secondary structures which ignore fine details of the stem and loop lengths and nesting. There may, in fact, be good biological reasons for doing this coarse-graining if one thinks that function or structure is not that sensitive to small changes in the full secondary structure. Another reason for our interest in this coarse-graining scheme comes from the work of Dingle *et al.* [31], who showed that the frequency with which non-coding or functional RNA abstract structure appears in the Rfam database [55,56] of non-coding or functional RNA could be remarkably well predicted over five orders of magnitude by the frequency by which these structures appear upon random sampling of sequences. This phenomenon illustrates how biases in the arrival of variation, which are mediated through the GP map, can dramatically affect evolutionary outcomes [15,26].

In coarse-grained GP maps, the value of the robustness is determined by the level of coarse-graining as well as by the underlying neutral set topologies. To study this problem, we will first derive analytic formulae which follow from the underlying graph theory. We then present numerical results on coarse-grained RNA GP maps and explain qualitative changes in robustness with phenotype coarse-graining. We conclude by deriving a critical transition probability that would be needed for two coarse-grained phenotypes to maintain ‘high’ robustness when coarse-grained together.

### 6.1. Robustness and transition probabilities for coarse-grained phenotypes

As before, we consider a GP map whose genotypes consist of sequences of length ℓ drawn from an alphabet of *k* characters. The genotype space is the Hamming graph *H*_ℓ,*k*_, and phenotype neutral sets are induced subgraphs of *H*_ℓ,*k*_. The *i*th phenotype’s neutral set *G*_*i*_ (assuming 1 ≤ *i* ≤ *N*_*p*_, where *N*_*p*_ is the total number of phenotypes) is an induced subgraph of *H*_ℓ,*k*_. Once again, we let *V*(*G*) denote the vertex set of a graph *G*, *E*(*G*) denote the edge set of *G*, and we additionally define *E*(*G*_*i*_, *G*_*j*_) = *E*_*H*_(*G*_*j*_, *G*_*i*_) to denote the set of edges induced in graph *H*_ℓ,*k*_ by union $V(G_{i})\cup V(G_{j})$ which are neither elements of *E*(*G*_*i*_) nor *E*(*G*_*j*_), where we have taken both *G*_*i*_ and *G*_*j*_ for *i* ≠ *j* to be induced subgraphs of *H*_ℓ,*k*_. Precisely, $E(G_{i},G_{j})=\{\{u,v\}\in H_{\ell ,k}|u\in V(G_{i})\wedge v\in V(G_{j})\}$.

As we have defined before, the robustness of the *i*th phenotype is defined as

6.1 $$\rho_{i}=\frac{2|E(G_{i})|}{\ell (k-1)|V(G_{i})|}.$$

In other words it is proportional to the ratio of edges to vertices in the neutral set graph. The transition probability that a single point mutation in the genotype leads to a change from phenotype *i* to phenotype *j* is defined by

6.2 $$\phi_{\;ji}=\frac{\left|E(G_{i},G_{j})\right|}{\ell (k-1)|V(G_{i})|},\;i\neq j,$$

which is again the average number of links per node between the two distinct neutral sets *i* and *j*. Note that *ϕ*_*ji*_|*V*(*G*_*i*_)| = *ϕ*_*ij*_|*V*(*G*_*j*_)|. If we define the diagonal terms *ϕ*_*ii*_ ≡ *ρ*_*i*_ then there needs to be an additional prefactor of 2 since the connections are between nodes of the same neutral set, and so must be counted twice.

#### 6.1.1. Robustness of coarse-grained phenotypes

We first derive a general formula for robustness of coarse-grained phenotypes. Let *S* be the set of phenotype indices that indicate which phenotypes are being coarse-grained into a new neutral set *G*_*S*_. The vertex set of *G*_*S*_ is the union of all vertices

6.3 $$V(G_{S})=\underset{s\in S}{⋃}V(G_{s}).$$

The edge set of *G*_*S*_ includes all edges in each individual neutral set as well as the edges joining the neutral sets

6.4 $$E(G_{S})=\left(\underset{s\in S}{⋃}E(G_{s})\right)\cup \left(\underset{(r,s)\in S}{⋃}E(G_{r},G_{s})\right),$$

where (*r*, *s*) ∈ *S* denotes an ordered pair of elements of *S* such that *r* > *s*. It follows that the robustness *ρ*_*S*_ of coarse-grained phenotype *S* is

6.5 $$\rho_{S}=\frac{2}{\ell (k-1)}\frac{\sum_{s\in S}|E(G_{s})|+\sum_{(r,s)\in S}|E(G_{r},G_{s})|}{\sum_{s\in S}|V(G_{s})|}.$$

Using equation (6.1) and the normalized phenotype frequency *f*_*i*_ = |*V*(*G*_*i*_)|/*k*^ℓ^, we can rewrite the coarse-grained robustness in terms of familiar biological parameters

6.6 $$\begin{array}{rl} \rho_{S} & =\frac{\sum_{s\in S}\rho_{s}f_{s}+2\sum_{(r,s)\in S}\phi_{rs}f_{s}}{\sum_{s\in S}f_{s}}\\ & =\frac{\sum_{s\in S}\sum_{r\in S}\phi_{rs}f_{s}}{\sum_{s\in S}f_{s}}, \end{array}$$

where in the last step we have used *ϕ*_*ss*_ = *ρ*_*s*_. It is easy to check that, if *S* = {1, 2, …, *N*_*p*_} is the set of *all* phenotypes, then $\sum_{1\leq s\leq N_{p}}\phi_{rs}f_{s}=f_{r}$ (given the definition of *ϕ*_*rs*_), so *ρ*_*S*_ = 1 as expected. The intuition behind equation (6.6) is that the robustness of the coarse-grained phenotype includes terms that come from the frequency-weighted sum of the robustnesses of the original phenotypes, as was the case for combining neutral components in equation (5.2), plus a term that adds in the contribution from transition probabilities between the combined phenotypes. If there are many transitions between them, then the combined robustness will be higher because these are now also classed as an additional contribution to robustness.

#### 6.1.2. Transition probabilities between coarse-grained phenotypes

We now calculate a general formula for transition probabilities between coarse-grained phenotypes. Let *S* and *T* be two non-overlapping sets of phenotype indices that indicate which phenotypes are being coarse-grained into two coarse-grained neutral sets *G*_*S*_ and *G*_*T*_, respectively. The set of edges *E*(*G*_*S*_, *G*_*T*_) joining *G*_*S*_ and *G*_*T*_ is the union of all sets of edges that adjoin every pair of (non-coarse-grained) phenotypes, where within each pair one element is picked from the constituent phenotypes of *S* and the other is picked from constituent phenotypes of *T*. It follows that

6.7 $$E(G_{S},G_{T})=\underset{s\in S}{⋃}\underset{t\in T}{⋃}E(G_{s},G_{t}).$$

It now follows that the transition probability *ϕ*_*TS*_ from the *S*th coarse-grained phenotype to the *T*th coarse-grained phenotype (assuming *S* and *T* have no overlap) is

$$\begin{array}{rl} \phi_{TS} & =\frac{\left|E(G_{S},G_{T})\right|}{\ell (k-1)|V(G_{S})|}\\ & =\frac{1}{\ell (k-1)}\frac{\sum_{s\in S}\sum_{t\in T}|E(G_{s},G_{t})|}{\sum_{s\in S}|V(G_{s})|} \end{array}$$

6.8 $$\;=\frac{\sum_{s\in S}\sum_{t\in T}\phi_{ts}f_{s}}{\sum_{s\in S}f_{s}}.$$

**Figure 5.**
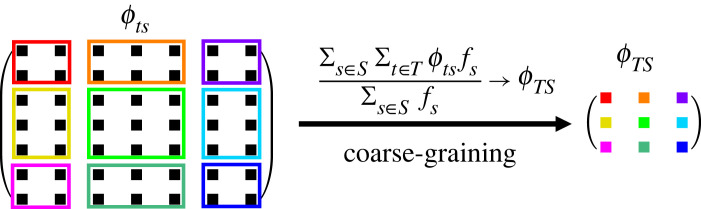
Schematic diagram of phenotype coarse-graining on the transition matrix *ϕ*_*ts*_ → *ϕ*_*TS*_, which includes transition probabilities (off-diagonals) and robustness (diagonals). If two non-overlapping sets of original phenotypes are coarse-grained into two new coarse-grained phenotypes *T* and *S*, then the transition probability from coarse-grained phenotype *S* to coarse-grained phenotype *T* is given by *ϕ*_*TS*_, calculated in equation (6.8), which involves taking a frequency-weighted sum over the transition probabilities *ϕ*_*ts*_ between the original non-coarse-grained phenotypes that comprise *T* and *S*.

We can now see from equations (6.6) and (6.8) that the coarse-graining procedure takes on the same functional form, which is represented graphically in figure 5.

**Figure 6.**
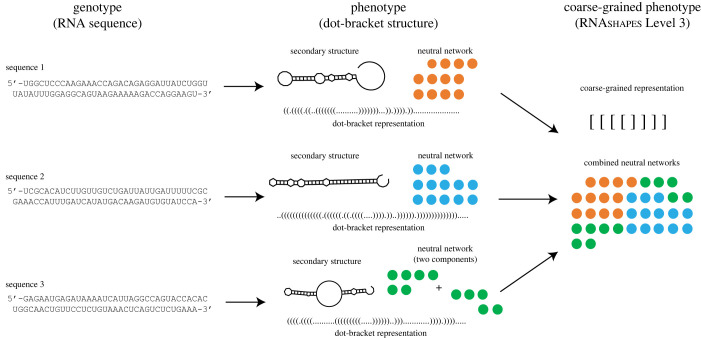
Demonstration of coarse-graining for RNA secondary structures. Genotypes (RNA sequences) map to secondary structure phenotypes represented as dot-bracket structures produced by ViennaRNA program [58]. The RNAshapes program [57] then progressively computes coarse-graining at different ‘levels ’, increasingly ignoring nesting of secondary structure topological features. In this figure, three example sequences of length *L* = 70 are shown to map to their dot-bracket phenotypes. A cartoon of a neutral set (not actual size) has been drawn for each phenotype; network properties like robustness can be calculated for each phenotype. At a higher level of coarse-graining (here, Level 3), multiple dot-bracket phenotypes all map onto the same coarse-grained phenotype. Accordingly, the coarse-grained phenotype has a neutral set comprising the individual neutral sets of the original phenotypes. Note that in practice there are many more secondary structures beyond those shown that map to this same coarse-grained phenotype; the analysis above is schematic.

**Figure 7.**
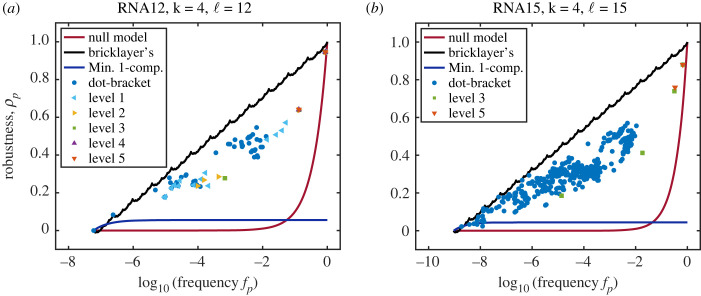
RNA abstract phenotype robustness plots for various levels of coarse-graining for (*a*) RNA12 and (*b*) RNA15 models. ‘Dot-bracket’ structures are the standard folded RNA obtained from the ViennaRNA program [58] folding results. The subsequent levels of coarse-grained structures are then obtained from the RNAshapes program [57]. Level 1 is the first abstracted (coarse-grained) phenotype, including one or more dot-bracket structures based on coarse-grained topology. Level 2 includes phenotypes that are further coarse-grained from Level 1; Level 3 includes phenotypes that are even further coarse-grained, etc. In the case of RNA12, Levels 4 and 5 are identical because the Level 4 phenotypes are already coarse-grained as much as possible. Also plotted are the bricklayer’s bound indicating the maximum possible robustness, the null model robustness (equation (1.2)), and the minimum robustness for a phenotype that contains only one component (equation (4.1)); this would be the robustness of a star graph [25].

The process of coarse-graining neutral components into a phenotype neutral set, handled in §5, is a specific case of the more general process of coarse-graining phenotype neutral sets, but in the former case, all transition probabilities between neutral components *ϕ*_*ji*_ = 0 since neutral components are not connected to each other, by definition. We now examine coarse-graining in the RNA secondary structure GP map. The RNAshapes program [57] merges RNA phenotypes at different ‘levels’ of coarse-graining by progressively ignoring more and more layers of detail in the stem and loop lengths and nesting structure of the folded RNA oligonucleotides. The ‘dot-bracket’ structure is the actual RNA secondary structure phenotype, obtained from the ViennaRNA program [58]. The dot-bracket structure is then provided to the RNAshapes program to produce the coarse-grained structures. Level 1 of coarse-graining ignores some details of the dot-bracket structure and combines similar phenotypes into the same abstract phenotype; the Level 2 structures include further coarse-graining, and so forth. There are five possible levels of coarse-graining. A schematic of the RNA coarse-graining process is shown in figure 6. In figure 7, we present results in which RNA secondary structure GP maps have robustness values calculated for these various levels of coarse-graining, performed using the RNAshapes tool.

Even though the systems in figure 7 are too small to show many Level 4 or 5 coarse-grained phenotypes, the overall trends are visible. The dot-bracket structures appear to be closest to the bricklayer’s graph maximum robustness curve. At the highest levels of coarse-graining (Level 4/5), abstract phenotypes are so densely packed with dot-bracket phenotypes that a substantial portion of the Hamming graph sequence space is covered by only a small number of abstract phenotypes. This leads to a percolation-like phenomenon that allows for highly coarse-grained, large-frequency phenotypes having high robustness as would be intuitively expected.

At lower levels of coarse-graining, however, we see a trend that we did not at first expect. It seemed to us reasonable to assume that coarse-graining dot-bracket phenotypes together, which increases the frequency, would simply ‘push’ the robustness parallel to the diagonal logarithm line. However, the data show that coarse-grained phenotypes with sufficiently small frequencies deviate further from the maximal possible robustness (the bricklayer’s graph bound) than the phenotypes that comprise them. This is because the transition probabilities between these phenotypes being coarse-grained are probably too low to provide adequate increase in robustness after coarse-graining. Note that, in contrast to robustness, transition probabilities are expected to scale as *ϕ*_*ij*_ ≈ *f*_*j*_ for RNA [15], as well as some other models [19] such as the HP model [41] and the polyomino model for protein quaternary structure [12,59]. In other words, the scaling of the transition probability versus frequency is no different from what would be expected from random assignment of GP pairs, so that these values are typically much lower than the robustness.

### 6.2. Critical threshold for the coarse-graining of phenotypes with high robustness

We now consider the example of coarse-graining two phenotypes and ask how much the transition probability between those phenotypes should be in order to keep them along the same diagonal robustness line parallel to the bricklayer’s graph bound. Recall that a phenotype’s neutral set *G*_*i*_ which contains *n* vertices has at most |*E*(*G*_*i*_)| = *S*_*k*_(*n*) edges, where *S*_*k*_(*n*) is once again the sums-of-digits function. We know that asymptotically *S*_*k*_(*n*) ∼ (*n*/2)log_*k*_ *n*, and a reasonable approximation to the maximum robustness is

6.9 $$\rho_{i}^{\max}=\frac{2S_{k}(n)}{n\ell (k-1)}\leq 1+\frac{\log_{k}f_{i}}{\ell }.$$

In the *high-robustness* asymptotic assumption, employed below, we assume that the robustness is near this bound and approximate it as *ρ*_*i*_ ≈ 1 + ℓ^−1^log_*k*_ *f*_*i*_.

For two phenotypes *p* and *q* that are being coarse-grained into a new phenotype *S*, we can use equation (6.6) to show that

6.10 $$\rho_{S}=\frac{\rho_{p}f_{p}+\rho_{q}f_{q}+2\phi_{qp}f_{p}}{f_{p}+f_{q}}.$$

Let us assume that the two phenotypes have robustness values that are displaced from the (asymptotic) optimal robustness curve by amounts Δ_*p*_ and Δ_*q*_

6.11 $$\rho_{p}=1+\frac{\log_{k}f_{p}}{\ell }-\Delta_{p}\;\mathrm{and}\;\rho_{q}=1+\frac{\log_{k}f_{q}}{\ell }-\Delta_{q}.$$

Substituting these approximations into equation (6.10), we have

6.12 $$\rho_{S}\approx 1-\frac{\;f_{p}\Delta_{p}+f_{q}\Delta_{q}}{f_{p}+f_{q}}+\frac{f_{p}\log_{k}f_{p}+f_{q}\log_{k}f_{q}+2\ell \phi_{qp}f_{p}}{\ell (f_{p}+f_{q})}.$$

We would intuitively expect two very robust phenotypes *p* and *q* that have dense connections to each other (i.e. relatively high values of *ϕ*_*qp*_*f*_*p*_ = *ϕ*_*pq*_*f*_*q*_) to deviate from the optimal robustness curve by Δ_*p*_ and Δ_*q*_. That is to say, we expect the robustness of the coarse-grained phenotype *S* to be bounded above by

6.13 $$\rho_{S}^{u}\approx 1+\frac{\log_{k}(f_{p}+f_{q})}{\ell }-\text{min}(\Delta_{p},\Delta_{q})$$

and bounded below by

6.14 $$\rho_{S}^{l}\approx 1+\frac{\log_{k}(f_{p}+f_{q})}{\ell }-\max(\Delta_{p},\Delta_{q}).$$

**Figure 8.**
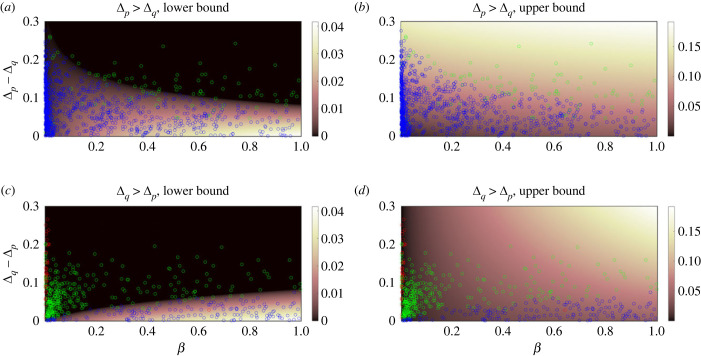
Plots of the (*a*,*c*) lower bounds and (*b*,*d*) upper bounds on *ϕ*_*qp*_ required for the coarse grained robustness to deviate from the maximum robustness curve by an amount that is at most max (Δ_*p*_, Δ_*q*_) and at least min (Δ_*p*_, Δ_*q*_). Contours are plotted versus the difference (*a*,*c*) Δ_*p*_ − Δ_*q*_ when Δ_*p*_ > Δ_*q*_ and (*c*,*d*) Δ_*q*_ − Δ_*p*_ when Δ_*p*_ < Δ_*q*_ on ordinate and versus the ratio of frequencies *β* ≡ *f*_*q*_/*f*_*p*_ with 0 < *β* ≤ 1 on the abscissa. 1187 phenotype pairs’ transition probabilities (blue) undershoot the lower bound, 423 transition probabilities (green) fall within the bounds derived above, and 43 transition probabilities (red) overshoot the upper bound.

The following inequalities provide bounds on the transition probability *ϕ*_*qp*_ in order for $\rho_{S}^{l}\leq \rho_{S}\leq \rho_{S}^{u}$ to be satisfied. The exact bounds are derived in appendix B and plotted in figure 8.

We find that, among all 1653 possible pairs of phenotypes in the RNA12 GP map, 1187 (71.8%) phenotype pairs’ transition probabilities undershoot the lower bound, which would lead to the coarse-grained phenotype deviating from the logarithmic upper bound (the approximation of the bricklayer’s bound) more than either of the two basic phenotypes. This means that, for most pairs of phenotypes, the number of edges connecting two random phenotypes is too low to pass the threshold required to obtain a coarse-grained robustness that is no less than the max possible robustness minus max (Δ_*p*_, Δ_*q*_). Four hundred and twenty-three transition probabilities fall within the bounds derived above. The remaining 43 transition probabilities overshoot the upper bound, thereby bringing the coarse-grained phenotype closer to the logarithmic upper bound. Moreover, 42 of those 43 pairs of phenotypes involve the non-folding phenotype, which is the highest occurring phenotype, with frequency 0.854, since most sequences in the RNA12 GP map do not have a folded structure as the minimum free energy structure. The frequency of 0.854 is higher than the percolation threshold of 1/[ℓ(*k* − 1)] = 1/36 ≈ 0.028, above which the neutral set contains a large component on the order of the system size. It is also higher than the giant component threshold 1 − *k*^−(1/(*k*−1))^ = 1 − 2^−2/3^ ≈ 0.37, above which the components of the neutral network coalesce into one large giant component that is (nearly) fully connected [19]. A giant component which occupies most of genotype space is more likely to share many edges with neutral networks of other phenotypes; as a result, the transition probabilities tend to be high, and coarse-graining another neutral network into the giant component simply forms an even larger, more robust giant component in most cases. Figure 8, along with the bounds, shows colour-coded plots of the transition probabilities which undershoot, overshoot and fall within the bounds.

It should be noted that, from a physical standpoint, it makes little sense to coarse-grain the non-folding phenotype along with a folding phenotype unless one were constructing, for instance, a coarse-grained ‘non-functional’ phenotype which incorporated phenotypes with low biological prevalence of functionality. Here, we entertain the above discussion only to offer physical intuition for why some transition probabilities cross the upper bound calculated earlier. In general, we expect that the intuition and theoretical insight gained from our formulation will be useful in the study of coarse-grained phenotypes, which is an active area in the field of GP maps [31,60].

## 7. Discussion

In this paper, we investigate maximally robust neutral sets known as bricklayer’s graphs. By applying concepts from coding theory as well as results from number theory on the sums-of-digits function, we analytically calculate the maximum phenotype robustness of a biological neutral set, a quantity which plays an important role in GP maps. We used numerical simulation to show, for the RNA sequence-to-secondary structure GP map and for the HP map for protein folding, that many neutral components have robustness that is near or achieves the upper bound.

We then derived a new property of sums of digits and used it to calculate a lower bound on the deviation of the robustness from the maximum bound when a neutral set is made up of independent neutral components. Similar bounds for the deviation from the maximum robustness that occur when phenotypes are coarse-grained together were also derived. By coarse-graining RNA secondary structures into abstract shapes [54], we demonstrated that our bounds provide intuition for trends observed in the behaviour of the robustness of coarse-grained abstract RNA shapes.

It remains an open question as to why GP maps generically have such high robustness. There are heuristic arguments based on constrained and unconstrained sites that help point in this direction [17,20,61] for systems such as RNA, and it would be interesting to explore how they link to our graph-theoretical approach. The bricklayer’s graph involves genotype networks that have precisely constrained genetic sites by construction. It is therefore perhaps surprising to see that many neutral components identified in §4 achieve the exact bricklayer’s bound, though it is possible that some of these graphs may not exactly be bricklayer’s graphs, but rather other small graphs that obtain the same bound. While combined phenotypes and neutral sets made up of multiple neutral components cannot exactly achieve this bound, it remains the case that their robustness is much closer (on a log scale) to this bricklayer’s bound than it is to a random-null model of uncorrelated phenotypes. Important ideas for future work include studying other GP maps, to see how close their robustness is to our theoretical maximum. In naturally occurring functional RNA, the mutational robustness is very close to that predicted by random sampling of genotypes for the GP map [26,36], which provides a neat example of detailed mathematical structure of the GP map being reflected in the living world. It would be extremely interesting to see if other biological systems exhibit mutational robustness that can also be predicted in this way.

Arguments from algorithmic information theory suggest that biological GP maps and other input–output maps share common underlying principles of organization [62]. This begs the question of how close the robustnesses of non-biological systems such as spin glasses [25], quantum circuits [63] and linear genetic programs [24] are to the maximum robustness we calculate here.

Another interesting direction of future work would be to better understand the spectral properties of bricklayer’s graphs. These may provide insight into population distributions and average robustness on long evolutionary time scales [40,42], allowing the exploration of relationships between mutational robustness and spectral properties.

## Figures and Tables

**Figure 9.**
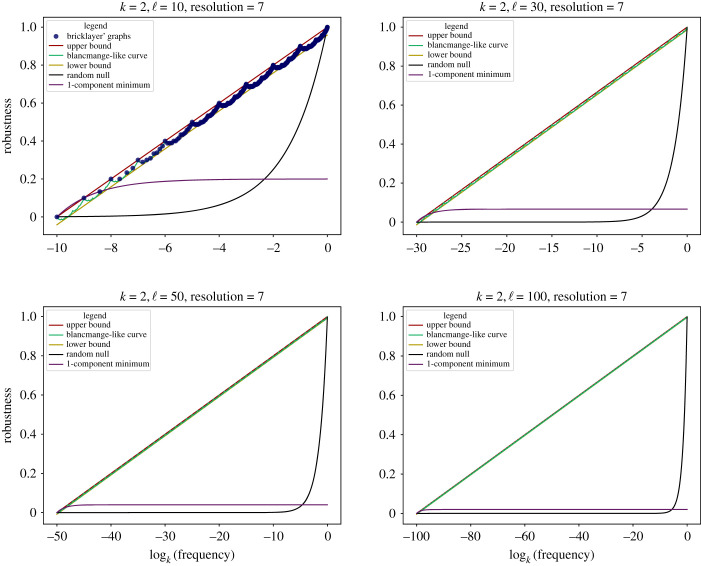
Plots of the continuous interpolation of the maximum robustness curve (bricklayer’s bound/blancmange-like curve), upper and lower bounds on the maximum robustness, the random null expectation robustness and the single neutral component minimum robustness, generated using RoBound Calculator [52] using *k* = 2 (corresponding to a binary alphabet, such as in the HP lattice protein model). Plots for ℓ = 10, 30, 50 and 100 are shown. The *k* = 2, ℓ = 10 plot also shows, as blue dots, the exact robustness of bricklayer’s graphs with an integer number of genotypes/vertices.

**Figure 10.**
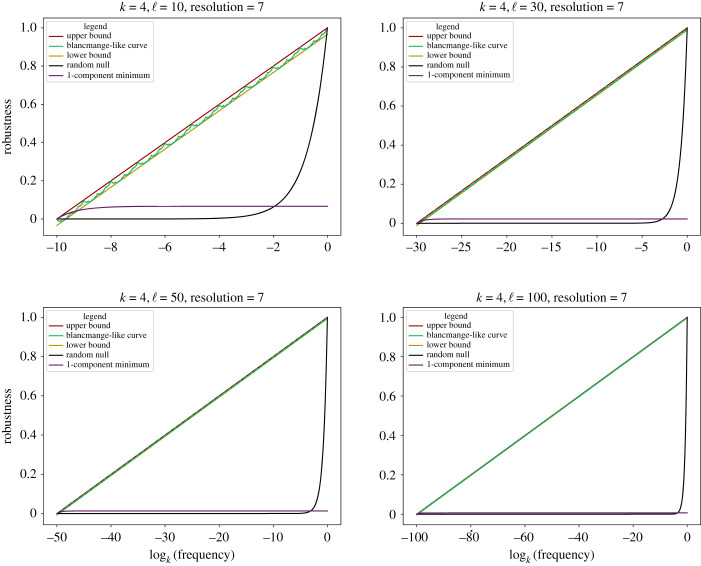
Plots of the continuous interpolation of the maximum robustness curve (bricklayer’s bound/blancmange-like curve), upper and lower bounds on the maximum robustness, the random null expectation robustness and the single neutral component minimum robustness, generated using RoBound Calculator [52] using *k* = 4 (corresponding to the RNA alphabet). Plots for ℓ = 10, 30, 50 and 100 are shown.

**Figure 11.**
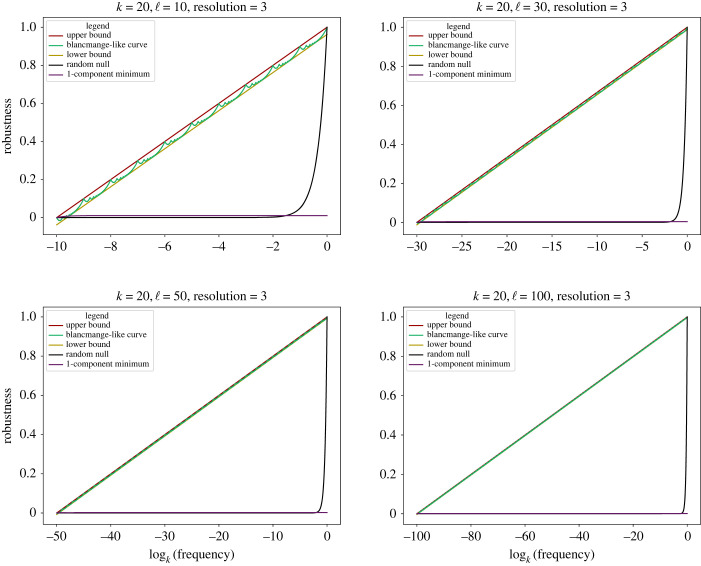
Plots of the continuous interpolation of the maximum robustness curve (bricklayer’s bound/blancmange-like curve), upper and lower bounds on the maximum robustness, the random null expectation robustness and the single neutral component minimum robustness, generated using RoBound Calculator [52] using *k* = 20 (corresponding to the number of standard amino acids found in proteins). Plots for ℓ = 10, 30, 50 and 100 are shown.

## Data Availability

We have introduced the web tool RoBound Calculator, a Google Colaboratory notebook which can generate, for specified ℓ and *k*, a continuous interpolation of the maximum robustness curve, tight upper and lower bounds on the maximum robustness curve, the exact robustnesses of bricklayer’s graphs comprising 1 to *k*^ℓ^ genotypes, the random null expectation of robustness and the minimum robustness curve for a single neutral component. The RoBound Calculator is available free of charge, with open-source code at the GitHub link in [52]. The data are available from the Dryad Digital Repository: https://datadryad.org/stash/dataset/doi:10.5061/dryad.sj3tx969f.

## Appendix A. Proofs of main text theorems

Theorem 3.1. *A bricklayer’s graph* *G*_*n*,*k*_(*V*, *E*) *with* *n* *vertices has* $\left|E|=S_{k}(n)=\sum_{i=0}^{n-1}s_{k}(i\right)$ *edges, where* *s*_*k*_(*i*) *is the sum of all digits in the base*-*k* *representation of the integer* *i*, *and* *S*_*k*_(*n*) *is the sums-of-digits function*.

Proof. To see this, let ℓ be the length of the input sequence so ℓ ≥ log_*k*_ *n*, and let (*x*_ℓ−1_(*n*), …, *x*_0_(*n*)) be the vector of integers containing the digits of the base-*k* representation of the integer *n* such that $n=\sum_{i=0}^{\ell -1}x_{i}(n)k^{i}$. Consider the bricklayer’s graph *G*_*n*−1,*k*_. When we add one more vertex such that $G_{n-1,k}\mapsto G_{n,k}$, we look at the base-*k* representation of *n*. An edge can be added if the base-*k* representation of *n* differs from the base-*k* representation of the neighbouring vertex by exactly one digit. Going through digit by digit, we see that the only allowed flips for the *i*th digits are $x_{i}(n)\mapsto \{0,\ldots ,x_{i}(n)-1\}$. This set has cardinality *x*_*i*_(*n*). Summing this over all digits, we find that the number of edges added to the graph *G*_*n*−1,*k*_ when adding an additional vertex *n* is the sum of digits of *n* in the base-*k* representation *s*_*k*_(*n*). Therefore, the total number of edges in *G*_*n*,*k*_ is $S_{k}(n)=\sum_{i=0}^{n-1}s_{k}(i)$. ▪

Theorem 5.1. *For* *k* *non-negative integers* {*n*_1_, *n*_2_, …, *n*_*k*_} *obeying* 0 ≤ *n*_1_ ≤ *n*_2_ ≤ · · · ≤ *n*_*k*_, *the following property of the sums-of-digits function holds*:

5.3 $$\sum_{i=1}^{k}S_{k}(n_{i})+\sum_{i=1}^{k-1}(k-i)n_{i}\leq S_{k}\left(\sum_{i=1}^{k}n_{i}\right)$$

Proof. Let $n=\sum_{i=1}^{k}n_{i}$, and choose ℓ such that *n*_*k*_ ≤ *k*^ℓ^. We must necessarily have that *n* ≤ *k n*_*k*_ ≤ *k*^ℓ+1^. Consider the Hamming graph *H*_ℓ+1,*k*_. Since $H_{\ell +1,k}=H_{\ell ,k}◻K_{k}$, we can decompose the edge set of *H*_ℓ+1,*k*_ into

A 1 $$E(H_{\ell +1,k})=\left(\overset{k}{\underset{i=1}{⋃}}E(H_{\ell ,k}^{\left(i\right)})\right)\cup \left(\underset{1\leq i<j\leq k}{⋃}E(H_{\ell ,k}^{\left(i\right)},H_{\ell ,k}^{\left(j\right)})\right),$$

where $H_{\ell ,k}^{\left(i\right)}$ is the Hamming graph consisting of the base-*k* representations of the integers whose (arbitrarily) first digit is *i* − 1, and $E(G_{1},G_{2})=\{\{u,v\}|u\in E(G_{1})\wedge v\in E(G_{2})\}$ is the set of edges in *H*_ℓ+1,*k*_ which join two subgraphs *G*_1_ and *G*_2_. Note that for 1 ≤ *i* ≤ *k*, we can construct a bricklayer’s graph $G_{n_{i},k}$ with *n*_*i*_ vertices that is a subgraph of the Hamming graph $H_{\ell ,k}^{\left(i\right)}$. By theorem 3.1, each bricklayer’s graph has size (number of edges) equal to *S*_*k*_(*n*_*i*_). Let us assume that each bricklayer’s graph has been constructed starting on the vertex such that its index’s first digit is *i* − 1, and all other digits are 0. This ensures that the $|E(G_{n_{i},k},G_{n_{j},k})|=n_{i}$ for *i* < *j* is maximal. The total number of edges in this graph *G*—i.e. the subgraph *G* of the Hamming graph *H*_ℓ+1,*k*_ induced by the vertex set V(G)= $\overset{k}{\underset{i=1}{⋃}}V(G_{n_{i},k})$—is given by the contributions from within the bricklayer’s graphs and the connections between them

$$\begin{array}{rl} \left|E(G)\right| & =\sum_{i=1}^{k}|E(G_{n_{i},k})|+\sum_{1\leq i<j\leq k}|E(G_{n_{i},k},G_{n_{j},k})|\\ & =\sum_{i=1}^{k}S_{k}(n_{i})+\sum_{1\leq i<j\leq k}n_{i}\\ & =\sum_{i=1}^{k}S_{k}(n_{i})+\sum_{i=1}^{k-1}\sum_{\;j=i+1}^{k}n_{i} \end{array}$$

A 2 $$\;=\sum_{i=1}^{k}S_{k}(n_{i})+\sum_{i=1}^{k-1}(k-i)n_{i}.$$

However, we also know that *G* has |*V*(*G*)| = *n* and, by theorem 3.1, size |*E*(*G*)| ≤ *S*_*k*_(*n*). Therefore,

A 3 $$|E(G)|=\sum_{i=1}^{k}S_{k}(n_{i})+\sum_{i=1}^{k-1}(k-i)n_{i}\leq S_{k}(n),$$

and this completes the proof. ▪

## Appendix B. Derivation of bounds on transition probability for coarse-graining of robustness

Starting with equation (6.13), we now perform a change of variables to *β* ≡ *f*_*q*_/*f*_*p*_ and *f*_*S*_ ≡ *f*_*p*_ + *f*_*q*_, so *f*_*p*_ = *f*_*S*_/(1 + *β*) and *f*_*q*_ = *f*_*S*_*β*/(1 + *β*). Without loss of generality, we can choose phenotype assignment such that *f*_*q*_ ≤ *f*_*p*_, i.e. 0 ≤ *β* ≤ 1. We now rewrite equation (6.13) as

B1 $$\begin{array}{rl} \rho_{S} & \approx 1-\frac{\Delta_{p}+\beta \Delta_{q}}{1+\beta }+\frac{1}{\ell (1+\beta )}\log_{k}\left(\frac{\;f_{S}}{1+\beta }\right)\\ & \;+\;\frac{\beta }{\ell (1+\beta )}\log_{k}\left(\frac{\;f_{S}\beta }{1+\beta }\right)+\frac{2\phi_{qp}}{1+\beta }\\ & =\left(1+\frac{\log_{k}f_{S}}{\ell }-\frac{\Delta_{p}+\beta \Delta_{q}}{1+\beta }\right)-\frac{\log_{k}(1+\beta )}{\ell }+\frac{\beta \log_{k}\beta }{\ell (1+\beta )}+\frac{2\phi_{qp}}{1+\beta }. \end{array}$$

The actual coarse-grained robustness deviates from the upper bound, equation (6.13), by

B2 $$\rho_{S}^{u}-\rho_{S}=\frac{\Delta_{p}+\beta \Delta_{q}}{1+\beta }-\text{min}(\Delta_{p},\Delta_{q})+\frac{\log_{k}(1+\beta )}{\ell }-\frac{\beta \log_{k}\beta }{\ell (1+\beta )}-\frac{2\phi_{qp}}{1+\beta },$$

and it deviates from the lower bound, equation (6.14), by

B3 $$\rho_{S}-\rho_{S}^{l}=\max(\Delta_{p},\Delta_{q})-\frac{\Delta_{p}+\beta \Delta_{q}}{1+\beta }-\frac{\log_{k}(1+\beta )}{\ell }+\frac{\beta \log_{k}\beta }{\ell (1+\beta )}+\frac{2\phi_{qp}}{1+\beta }$$

In order for the actual coarse-grained robustness *ρ*_*S*_ to fall within the bounds of the interval $\left[\rho_{S}^{l},\rho_{S}^{u}\right]$, for Δ_*p*_ > Δ_*q*_, and with the constraint 0 ≤ *ϕ*_*qp*_ ≤ 1, we have

B4 $$\begin{array}{rl} & \max\left(0,\frac{1}{2}\left[\frac{(1+\beta )\log_{k}(1+\beta )-\beta \log_{k}\beta }{\ell }-\beta (\Delta_{p}-\Delta_{q})\right]\right)\leq \phi_{qp}\\ & \;\leq \text{min}\left(1,\frac{1}{2}\left[\frac{(1+\beta )\log_{k}(1+\beta )-\beta \log_{k}\beta }{\ell }+(\Delta_{p}-\Delta_{q})\right]\right), \end{array}$$

and for Δ_*p*_ < Δ_*q*_, with the constraint 0 ≤ *ϕ*_*qp*_ ≤ 1, we have

B5 $$\begin{array}{rl} & \max\left(0,\frac{1}{2}\left[\frac{(1+\beta )\log_{k}(1+\beta )-\beta \log_{k}\beta }{\ell }-(\Delta_{q}-\Delta_{p})\right]\right)\leq \phi_{qp}\\ & \;\leq \text{min}\left(1,\frac{1}{2}\left[\frac{(1+\beta )\log_{k}(1+\beta )-\beta \log_{k}\beta }{\ell }+\beta (\Delta_{q}-\Delta_{p})\right]\right). \end{array}$$

Collectively, we can write

B6 $$\begin{array}{rl} & \max(0,\frac{1}{2}[\frac{(1+\beta )\log_{k}(1+\beta )-\beta \log_{k}\beta }{\ell }+\Delta_{p}\\ & \;+\beta \Delta_{q}-(1+\beta )\max(\Delta_{p},\Delta_{q})])\leq \phi_{qp}\\ & \;\leq \text{min}(1,\frac{1}{2}[\frac{(1+\beta )\log_{k}(1+\beta )-\beta \log_{k}\beta }{\ell }\\ & \;+\Delta_{p}+\beta \Delta_{q}-(1+\beta )\text{min}(\Delta_{p},\Delta_{q})]). \end{array}$$

The lower and upper bounds on *ϕ*_*qp*_ for the cases Δ_*p*_ > Δ_*q*_ and Δ_*p*_ < Δ_*q*_ are plotted in figure 8. For Δ_*p*_ = Δ_*q*_, the range $\left[\rho_{S}^{l},\rho_{S}^{u}\right]$ collapses to a single value, and the constraint on *ϕ*_*qp*_ becomes an equality

B7 $$\phi_{qp}=\frac{1}{2}\left[\frac{(1+\beta )\log_{k}(1+\beta )-\beta \log_{k}\beta }{\ell }\right].$$

## Appendix C. Example plots using the RoBound calculator web tool

In this section of the appendix, we show representative plots generated from our web tool RoBound Calculator [52], which generates—provided ℓ, *k* and a resolution value—the continuous interpolation of the maximum robustness curve (bricklayer’s bound/blancmange-like curve), upper and lower bounds on the maximum robustness, the random null expectation robustness, the single neutral component minimum robustness and, if desired, the exact robustnesses of bricklayer’s graphs with an integer-valued number of nodes. The examples we provide are for sequences lengths ℓ = 10, 30, 50 and 100 for alphabet sizes *k* = 2 (corresponding to the HP lattice protein model, in figure 9), *k* = 4 (corresponding to the RNA alphabet, in figure 10), and *k* = 20 (corresponding to the number of amino acids in proteins, in figure 11). These plots help illustrate how for larger *k* and ℓ, the upper and lower bounds from equation (3.8) become tighter, so that the simple $\rho_{p}^{\max}\approx \log_{k}(n_{p})/\ell =1+\log_{k}(f_{p})/\ell$ form provides good approximation to the maximum robustness.
